# Cost-effectiveness analysis of trastuzumab deruxtecan in patients with HER2-low advanced breast cancer based on DESTINY-Breast04

**DOI:** 10.3389/fpubh.2023.1049947

**Published:** 2023-06-30

**Authors:** Mei Zhan, Zijia Huang, Ting Xu, Xinyi Xu, Hanrui Zheng, Fengbo Wu

**Affiliations:** ^1^Department of Pharmacy, West China Hospital, Sichuan University, Chengdu, China; ^2^West China School of Pharmacy, Sichuan University, Chengdu, China

**Keywords:** cost-effectiveness, breast cancer, HER2-low, trastuzumab deruxtecan, chemotherapy

## Abstract

**Background and purpose:**

Breast cancer is a rapidly raising healthcare problem worldwide. DESTINY-Breast04 demonstrated that trastuzumab deruxtecan (T-Dxd) had a survival advantage comparing to the physician's choice of chemotherapy for patients with HER2-low metastatic breast cancer. But at the same time, this expensive novel treatment also brought an economic burden. This study assessed the cost-effectiveness of T-Dxd based on results of DESTINY-Breast04 from the perspective of Chinese healthcare system.

**Materials and methods:**

A three-state partitioned-survival model [progression-free survival (PFS), progressive disease (PD) and death] based on data from DESTINY-Breast04 and Chinese healthcare system was used to estimate the incremental cost-effectiveness ratio (ICER) of T-Dxd vs. the physician's choice of chemotherapy for HER2-low metastatic breast cancer. Costs, quality-adjusted life-years (QALYs) and the ICER in terms of 2022 US$ per QALY gained were calculated for both hormone receptor–positive cohort and all patients. One-way and probabilistic sensitivity analyses were performed to assess the model robustness.

**Results:**

Compared with the physician's choice of chemotherapy, T-Dxd increased costs by $104,168.30, while gaining 0.31 QALYs, resulting in an ICER of $336,026.77 per QALY in all patients. The costs of T-Dxd and the utility of PFS were the crucial factors in determining the ICER. In the hormone receptor–positive cohort, the ICER was lower than that in all patients, with the ICER of $274,905.72 per QALY. The ICER was much higher than the commonly accepted willingness-to-pay threshold ($357,96.83 per QALY).

**Conclusion:**

T-Dxd as second- or subsequent-line treatment is not a cost-effective treatment option for HER2-low metastatic breast cancer from the perspective of the Chinese healthcare system.

## 1. Introduction

The burden of breast cancer is increasing rapidly. In 2020, there was an estimated 2.26 million new cases of breast cancer, making it the most commonly diagnosed cancer globally, surpassing even lung cancer. Breast cancer also created 684,996 deaths worldwide, ranking fifth among all cancer-related deaths (1). The age-standardized incidence and mortality rate of breast cancer have significantly increased in China during the past decade, putting a great burden on Chinese healthcare and economic system (2). Human epidermal growth factor receptor 2 (HER2)-low breast cancer, defined as HER2 immunohistochemistry (IHC) 1+ or IHC 2+ and insituhybridization (ISH)-negative, accounts for 40–50% of all breast cancers (3, 4). Previous HER2-targeted therapies remarkably improved clinical outcomes of HER2 positive breast cancer, but have failed to provide prognosis benefit in patients with HER2-low breast cancer. There is limited treatment option for progressed HER2-low breast cancer refractory to standard treatment, and patients often have to receive palliative chemotherapy. Therefore, creating effective new treatments for HER2-low breast cancer is of great clinical significance (5).

Trastuzumab deruxtecan (T-Dxd) is an antibody–drug conjugate (ADC) composed of trastuzumab and a topoisomerase I inhibitor through a tetrapeptide-based cleavable linker (6). Unlike many other HER2-targeted therapies, T-Dxd is also effective in HER2-low breast cancer due to its bystander effect (7, 8). The superiority of T-Dxd over traditional single-agent chemotherapy in patients with HER2-low breast cancer who had received one or two previous lines of treatment was demonstrated in DESTINY-Breast04 (9). Based on DESTINY-Breast04, the US Food and Drug Administration approved T-Dxd for patients with unresectable or metastatic HER2-low breast cancer who have received prior chemotherapy in the metastatic setting. The National Comprehensive Cancer Network (NCCN) also recommended T-Dxd as the preferred second-line therapy for HER2-low breast cancer (10).

However, while T-Dxd demonstrated survival advantage in DESTINY-Breast04, it is extremely expensive for both patients and insurance payers. As such, we sought to evaluate the cost-effectiveness of T-Dxd for advanced HER2-low breast cancer from the Chinese healthcare system perspective.

## 2. Methods

### 2.1. Patients and treatment

In the base case analysis, a hypothetical cohort was generated using the clinical information collected from DESTINY-Breast04 (9). The trial included a total of 557 HER2-low metastatic breast cancer patients, of whom 373 were randomly assigned to receive T-Dxd 5.4 mg/kg every 3 weeks (T-Dxd group) while 184 were assigned to the physician's choice of chemotherapy (chemotherapy group) when their breast cancer progressed after one or two previous lines of chemotherapy. 331 (88.7%) T-Dxd group patients and 163 (88.6%) chemotherapy group patients, respectively, were qualified for the hormone receptor–positive cohort. Treatment for chemotherapy group comprised of five regimens: capecitabine (20.1%), eribulin (51.1%), gemcitabine (10.3%), paclitaxel (8.2%), or nab-paclitaxel (10.3%). Overall survival (OS) and progression-free survival (PFS) were evaluated in the hormone receptor–positive cohort and in all patients.

### 2.2. Model structure and assumptions

**Figure 1:**
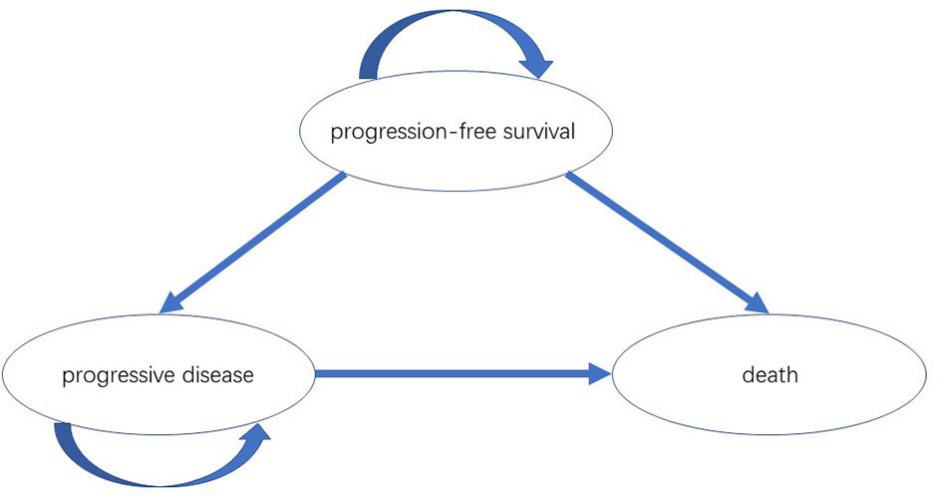
The partitioned-survival model simulated three health states: PFS, PD and death. PFS, progression-free survival; PD, progressive disease.

A partitioned-survival model was constructed by Treeage Pro Suite 2019 (Treeage Software, Inc., MA, USA) from the perspective of the Chinese healthcare system. The model included three mutually exclusive health states: PFS, progressive disease (PD) and death. The initial state was assumed to be PFS, and patients could remain in the PFS state or move to PD or death state during each cycle (Figure 1). We assumed that the cycle length was 1-month based on the time span of disease duration and progression. Patients with metastatic HER2-low breast cancer refractory to standard therapies have poor prognosis; the median overall survival ranged from 11.1 to 29.4 months (8, 9, 11). The population in the PSM model had received one or two previous lines of chemotherapy, and the median overall survival in DESTINY-Breast04 was less than two years (9). Therefore, a 5-year time horizon was selected for the model. The annual discount rates for costs and outcomes were set at 5% as recommended by guidelines, and discount rates of 0 and 8% were explored in scenario analyses (12). The threshold of willingness to pay (WTP) was assumed to be three times the Chinese per Gross Domestic Product per capita (GDP) according to WHO guideline (13). As a result, $357,96.83/quality-adjusted life-year (QALY) was set according to per capita GDP of China 2021 released by National Bureau of Statistics. All costs were converted into US dollars, with an exchange rate of $1 = ¥6.7863 (17 Aug 2022).

### 2.3. Clinical parameters from DESTINY-Breast04

Clinical data on efficacy and safety were obtained from DESTINY-Breast04. Survival parameters were obtained by digitizing the Kaplan– Meier (KM) curve (OS, PFS) of DESTINY-Breast04. Individual patient data were reconstructed using the method described by Guyot et al. (14). KM curves up to the end of follow-up period were followed by simulative curves generated from best-fit parametric distributions. Different parameter distributions (Exponential, Gamma, Gen gamma, Gompertz, Weibull, Log-logistic, Log-normal) were applied to fit the reconstructed OS and PFS curves. The best-fit parametric distributions were selected based on Akaike information criterion (AIC), Bayesian information criterion (BIC) and visual inspection. The IC values for all models were shown in Table 1.

**Table 1:** Results of the fit to the observed data.

	**Hormone receptor–positive cohort**								**All patients**							
	**OS of T-DXd**		**PFS of T-DXd**		**OS of chemotherapy**		**PFS of chemotherapy**		**OS of T-DXd**		**PFS of T-DXd**		**OS of chemotherapy**		**PFS of chemotherapy**	
	**AIC**	**BIC**	**AIC**	**BIC**	**AIC**	**BIC**	**AIC**	**BIC**	**AIC**	**BIC**	**AIC**	**BIC**	**AIC**	**BIC**	**AIC**	**BIC**
Exponential	1,149.75	1,153.55	1,493.30	1,497.10	647.67	650.76	618.79	621.88	1,313.07	1,316.99	1,755.62	1,759.54	730.71	733.93	725.48	728.69
Gamma	1,124.76	1,132.36	1,479.33	1,486.94	636.17	642.36	613.11	619.30	1,276.44	1,284.28	1,742.46	1,750.30	709.42	715.85	717.89	724.32
Gen gamma	1,122.53	1,133.93	1,477.94	1,489.35	637.56	646.85	601.61	610.89	1,278.18	1,289.94	1,742.19	1,753.95	711.28	720.92	703.71	713.36
Gompertz	1,121.52	1,129.12	1,489.99	1,497.60	638.57	644.75	620.56	626.74	1,284.91	1,292.76	1,751.28	1,759.12	719.06	725.49	727.20	733.63
Weibull	1,121.87	1,129.48	1,481.73	1,489.33	635.57	641.76	615.93	622.12	1,276.28	1,284.13	1,744.41	1,752.25	710.68	717.11	721.55	727.98
Log-logistic	1,125.70	1,133.31	1,478.45	1,486.05	636.57	642.75	606.70	612.88	1,276.86	1,284.70	1,745.00	1,752.84	708.48	714.91	709.49	715.92
Log-normal	1,143.33	1,150.94	1,478.04	1,485.64	645.34	651.53	601.35	607.54	1,284.34	1,292.18	1,744.78	1,752.63	712.74	719.17	703.39	709.82

AIC, Akaike information criterion; BIC, Bayesian Information Criterion; OS, Overall Survival; PFS, Progression-Free Survival; T-DXd, Trastuzumab Deruxtecan.

**Figure 2:**
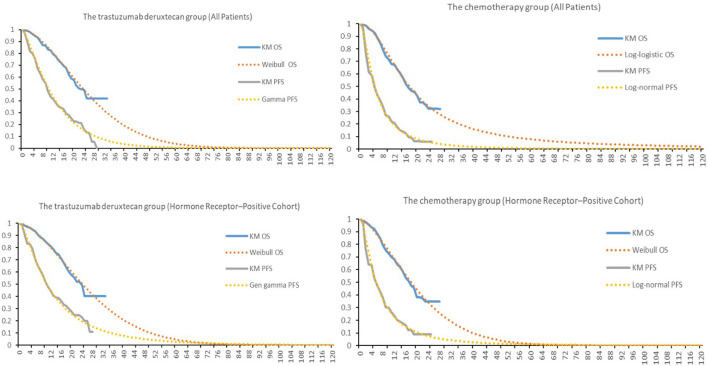
Kaplan-Meier survival for the trastuzumab deruxtecan and chemotherapy groups in DESTINY-Breast04 and the fitting curves. OS, overall survival; PFS, progression-free survival.

In the hormone receptor–positive cohort, Weibull distribution was selected to fit the KM curves for OS of both T-Dxd and chemotherapy group; for PFS, Gen gamma and Log-normal distribution were chosen for T-Dxd and chemotherapy group, respectively. Among all patients, Weibull distribution and Log-logistic distribution were found to fit the OS curve of the T-Dxd and the chemotherapy group, respectively; Gamma distributions and Log-normal distributions were selected to fit the PFS curve of the T-Dxd and the chemotherapy group, respectively. The original and the fitting curves were shown in Figure 2.

The incidence of adverse events (AEs) required to estimate the management cost of AEs was obtained from DESTINY-Breast04, more details ware shown Table 2. As quality-of-life data was not collected in DESTINY-Breast04, health state utility scores were derived from previously published literature. The utility values of PFS state, PD state and death were 0.843, 0.60 and 0, respectively (15).

**Table 2:** Clinical information based on DESTINY-Breast04.

**Variables**	**T-DXd**	**Chemotherapy**
**OS (months)**		
All patients	23.4	16.8
Hormone receptor–positive cohort	23.9	17.5
**PFS (months)**		
All patients	9.9	5.1
Hormone receptor–positive cohort	10.1	5.4
**Probability of grade 3/4 AEs**		
Neutropenia	13.70%	40.70%
Anemia	8.10%	4.70%
Thrombocytopenia	5.10%	0.60%
Leukopenia	6.50%	19.20%
Nausea	4.60%	0.00%
Vomiting	1.30%	0.00%
Diarrhea	1.10%	1.70%
Increased aminotransferase levels	3.20%	8.10%
Fatigue	7.50%	4.70%
Decreased appetite	2.40%	1.20%

OS, Overall Survival; PFS, Progression-Free Survival; T-DXd, Trastuzumab Deruxtecan.

### 2.4. Cost estimates

Direct medical costs consisted of drug treatment costs, AEs treatment costs, follow-up costs, hospital service costs, and best supportive care (BSC) costs, were estimated from the perspective of the Chinese healthcare system. Resource costs except for the drug treatment costs were obtained from Chinese studies.

Destiny-break04 did not provide a subsequent treatment plan for patients whose diseases progressed on T-Dxd or physician's choice of chemotherapy; according to the guideline, BSC is recommended for these patients as they have already received two lines of therapy (16). Costs related to subsequent BSC were derived from published literatures (17). The dosages of chemotherapy agents and T-Dxd were calculated based on standard human body surface area of 1.72 m^2^ and a standard female bodyweight of 55 kg, respectively (18). Although T-Dxd is yet to be approved for Chinese market, it became available in Hainan's Boao Lecheng International Medical Tourism Pilot Zone in February 2022. For this study, the price for T-Dxd in Chinese market was set with reference to the marketing price of T-Dxd in Boao. Prices of other drugs used in this study were calculated based on the median winning prices of the bid-winning products on https://www.yaozh.com/.

DESTINY-Breast04 reported data on incidences of adverse events (AEs). Only the costs related to managing grade 3 or higher AEs were included for this study; grade 1–2 AEs were considered manageable within standard patient monitoring. The costs of managing grade 3–5 AEs were derived from previously published economic studies (18–23). Detailed information was shown in Table 3.

**Table 3:** Base-case model inputs.

**Parameter**	**Value**
**Cost**	
T-DXd per 100 mg	2,431.37
Capecitabine per 0.5 g tablet	12.11
Paclitaxel	37.79
Nab-paclitaxel (per 100 mg)	114.94
Hospitalization per cycle	57.43
Post-progression per cycle	1,886.67
Follow-up per cycle	48.00
**SAE management cost per event**	
Neutropenia	547.50
Anemia	607.06
Thrombocytopenia	193.50
Leukopenia	104.95
Nausea	39.60
Vomiting	39.60
Diarrhea	44.30
Increased aminotransferase levels	68.30
Fatigue	131.78
Decreased appetite	115.40
**Utilities**	
PFS	0.843
PD	0.6
Discount rate	5%

PD, progressive disease; PFS, Progression-Free Survival; SAE, Serious Adverse Event; T-DXd: Trastuzumab Deruxtecan.

### 2.5. Sensitivity analysis

One-way sensitivity analysis and probabilistic sensitivity analyses (PSA) were performed to examine the potential influence on the results. In one-way sensitivity analysis, the most parameters of costs and utilities were varied at a range of ± 20% of their baseline value, and the range of discount rate was from 0 to 8%. Since T-Dxd has not been approved in Chinese Mainland, the price of trastuzumab deruxtecan may decrease sharply in the future. Therefore, the minimum cost of T-Dxd was set to a 50% decrement from the baseline value. The One-way sensitivity analysis results were presented in a tornado diagram. A PSA was performed by using Monte Carlo simulation of 1,000 iterations to assess the robustness of the estimated cost-effectiveness ratio. Gamma and Beta distributions were adopted for costs and utilities, respectively. The results of the PSA were represented by an acceptable curve and incremental cost-effectiveness scatter plot.

## 3. Results

### 3.1. Base-case analysis

In the base-case analysis, among all patients, the total cost was $145,887.58 for the T-Dxd group and $41,719.28 for the chemotherapy group. The overall QALYs in the T-Dxd group were higher than that in the chemotherapy group (1.57 QALYs vs. 1.26 QALYs). The incremental cost-effectiveness ratio (ICER) was $336,026.77 per QALY, which was more than 9 times the WTP threshold for cost-effectiveness ($357,96.83 per QALY in China). In the hormone receptor–positive cohort, the T-Dxd group comprised even higher QALY. The T-Dxd group cost $118,209.46 more than the chemotherapy group while providing additional 0.43 QALYs, leading to an ICER of $274,905.72 per QALY in the hormone receptor–positive cohort. The details are listed in Table 4.

**Table 4:** Base-case cost-effectiveness analysis results.

**Subgroups and strategies**	**Total population**		**Hormone receptor–positive cohort**	
	**T-DXd**	**Chemotherapy**	**T-DXd**	**Chemotherapy**
**Costs ($)**				
PFS state ($)	123,002.03	15,022.56	135,342.29	16,255.62
PD state ($)	22,885.55	26,696.72	20,636.88	21,514.07
Total Cost ($)	145,887.58	41,719.28	155,979.16	37,769.70
Incremental costs ($)	104,168.30		118,209.46	
**Effectiveness (QALYs)**				
PFS state (QALYs)	0.96	0.55	1.08	0.63
PD state (QALYs)	0.61	0.71	0.55	0.57
Total effectiveness (QALYs)	1.57	1.26	1.63	1.20
Incremental effectiveness (QALYs)	0.31		0.43	
ICERs compared with PC alone ($/QALY)	336,026.77		274,905.72	

ICER, Incremental Cost Effectiveness Ratios; PD, progressive disease; PFS, Progression-Free Survival; QALY, Quality-adjusted Life Year; T-DXd, Trastuzumab Deruxtecan.

### 3.2. Sensitivity analysis

**Figure 3:**
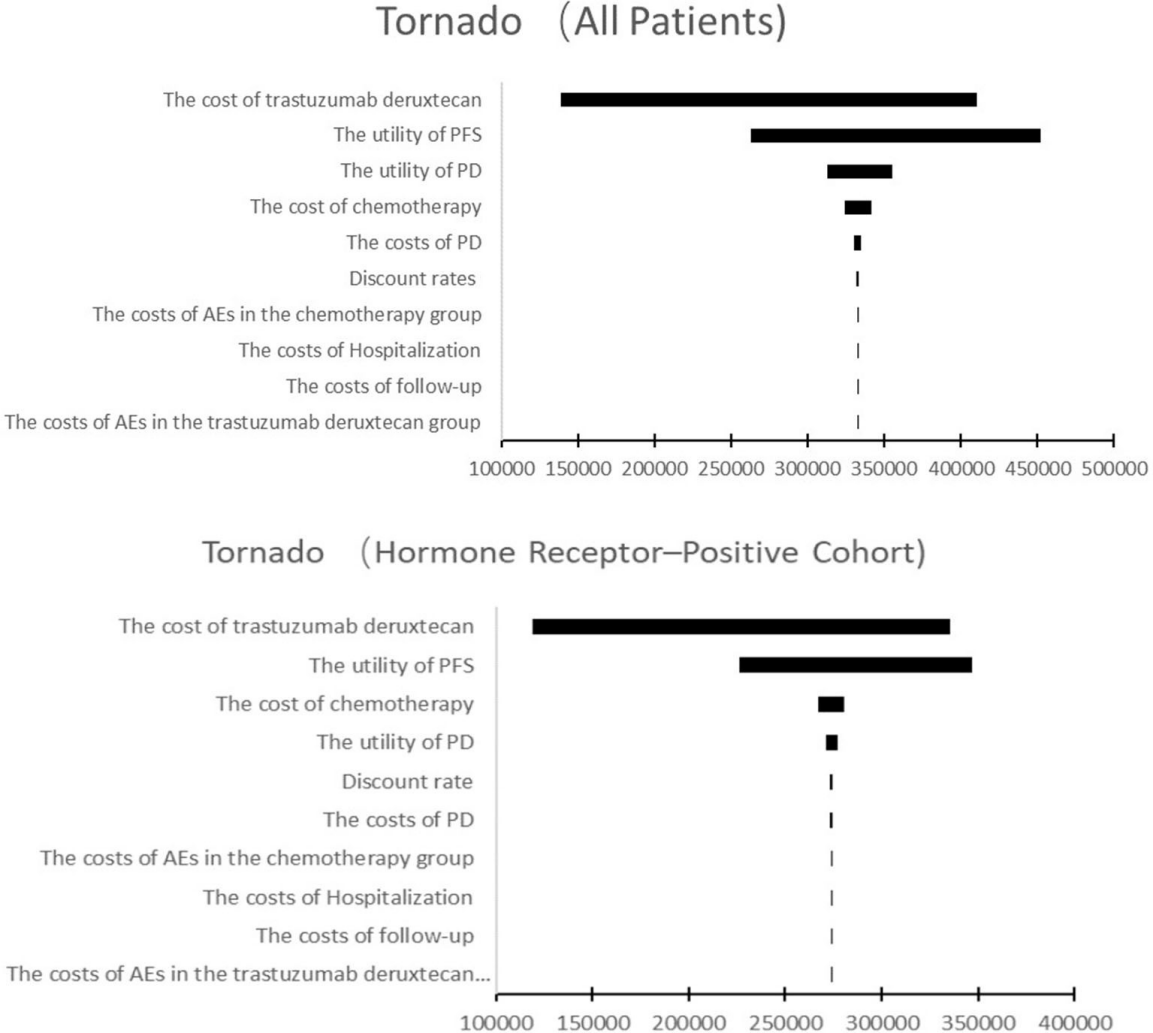
Tornado diagram of one-way sensitivity analysis. This summarizes the results of one-way sensitivity analysis, listing influential parameters in descending order according to their effect on the ICER over the variation of each parameter value. PFS, progression-free survival; PD, progressive disease; AE, adverse event.

The results of the one-way sensitivity analysis were shown in Figure 3. In both the hormone receptor–positive cohort and all patients, the cost of T-Dxd and the utility of PFS were the most influential factors on the results. In addition, the cost of chemotherapy and the utility of PD had moderate impact on ICER. Other parameters such as discount rate, costs of PD, AEs, hospitalization and follow-up had minor impact on the robustness of the cost-effectiveness analysis. More details were shown in Figure 3.

**Figure 4:**
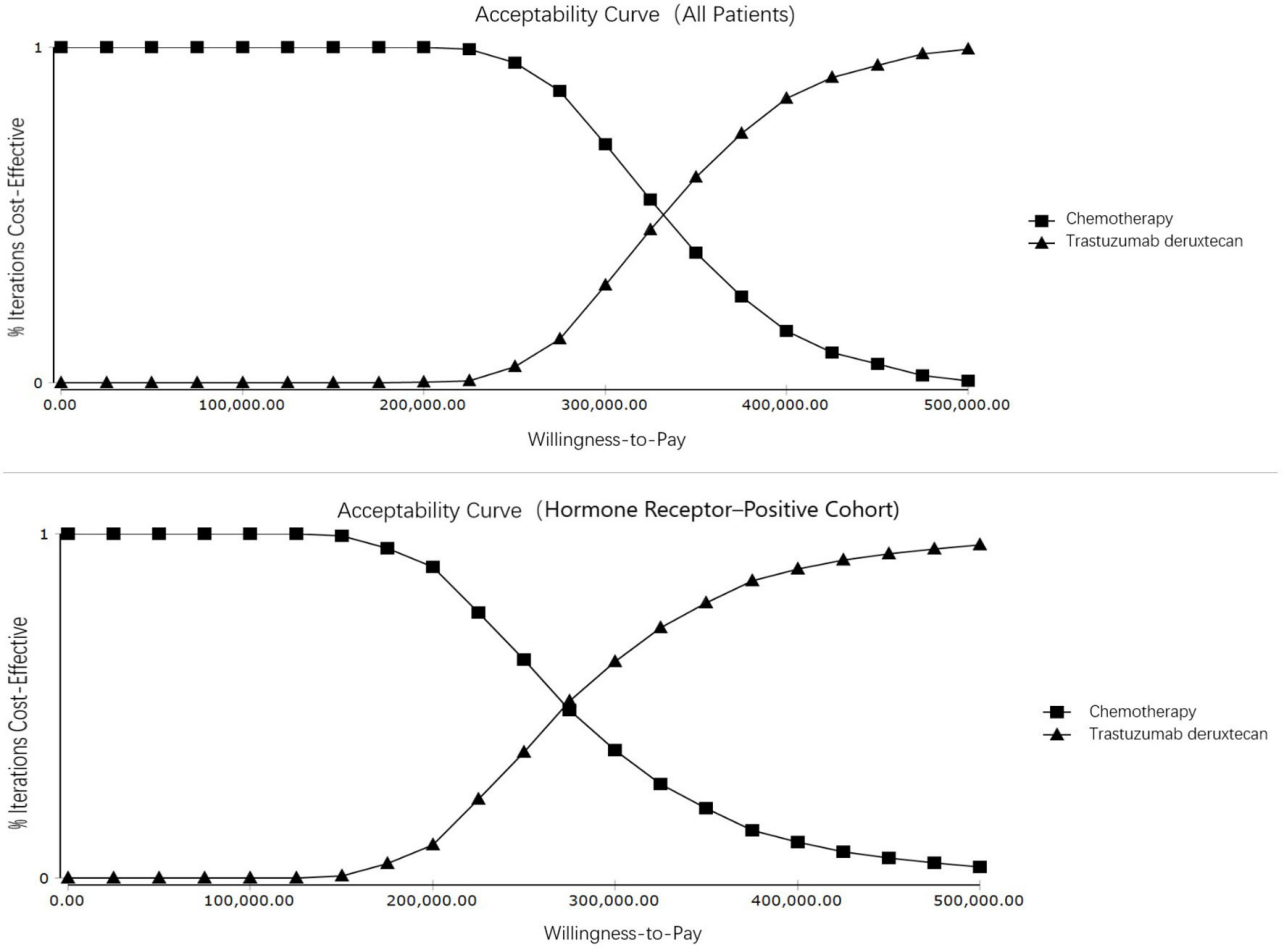
Cost-effectiveness acceptability curves. Cost-effectiveness acceptability curves show the probability of each treatment strategy being cost-effective at different willingness-to-pay thresholds.

**Figure 5:**
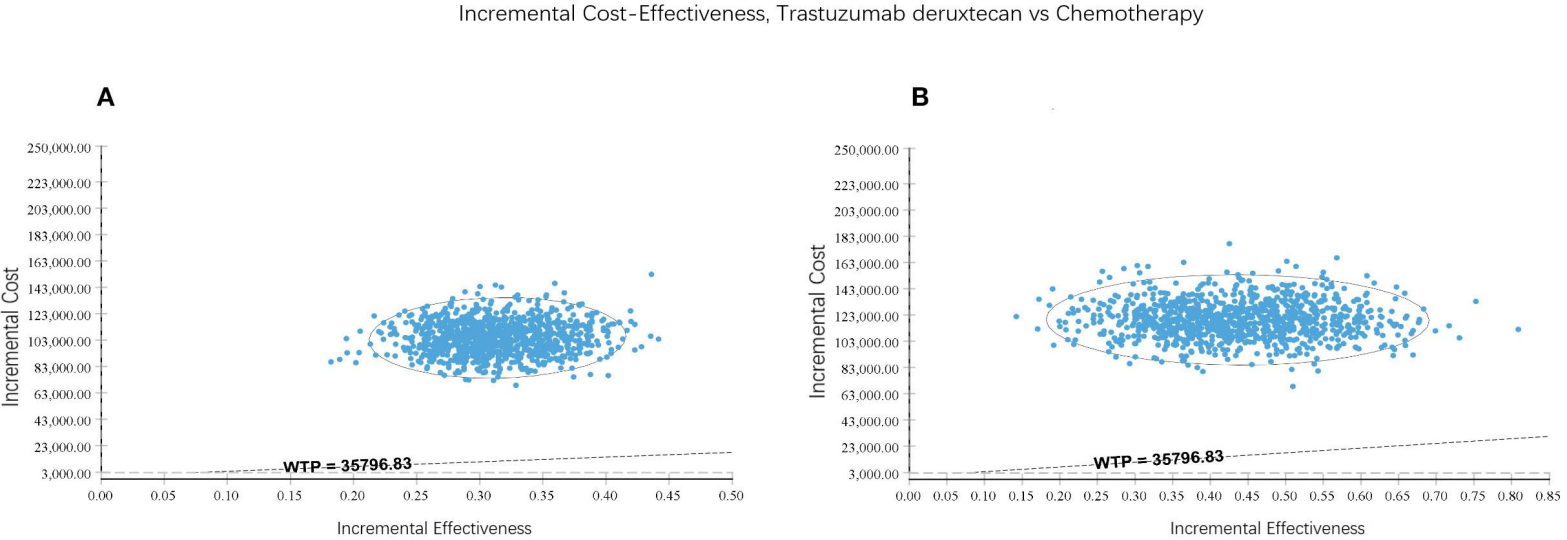
Incremental cost-effectiveness scatter plot reflected the variation and concentration of the incremental cost-effectiveness ratio values in probabilistic sensitivity analysis. **(A)** All patients. **(B)** Hormone receptor-positive cohort.

T-Dxd would not be cost-effective unless the threshold of the CEA sharply raise to about $170,000–$225,000 per QALY (Figure 4), which seems impossible as China's GDP cannot reach this level in the short term. The PSA suggested that compared with chemotherapy, the probability of T-Dxd being cost-effective was 0% at the WTP threshold of $35,796.83/QALY in both all patients and the hormone receptor–positive cohort (Figure 5). The results of PSA demonstrated that the T-Dxd had no economic advantage over the traditional chemotherapy in China in the near future.

## 4. Discussion

DESTINY serial studies were launched since the approval of T-Dxd. DESTINY-Breast−02, 03 and 04 studies discovered positive results in T-Dxd groups, changing treatment paradigms in breast cancer (9, 24, 25). As a novel therapy, T-Dxd was associated with high economic burden; therefore, pharmacoeconomic research based on DESTINY trials was warranted to evaluate its cost-effectiveness (26–29). Previously, Zhu et al. conducted a Markov decision-analytic model to evaluate the cost-effectiveness of T-DXd for HER2-low metastatic breast cancer in the United States; their study demonstrated that T-DXd was not cost-effective for patients with HER2-low advanced breast cancer comparing to chemotherapy in the United States. However, by December 2022, there has been no pharmacoeconomic evaluation based on DESTINY-Breast04 from the perspective of Chinese healthcare system. In this study, we proved that T-Dxd was not cost-effective for advanced HER2-low breast cancer compared with chemotherapy from the perspective of Chinese healthcare system using a three-state partitioned-survival model. The price of T-Dxd had highest impact on the ICER, which also aligns with the result from Zhu et al.

In 2013, The State Council officially approved the establishment of Hainan Boao Lecheng International Medical Tourism Pilot Zone, making Boao Lecheng the only area in mainland China that can market drugs that have been approved abroad but not yet marketed in mainland China. The price of T-Dxd was set at the marketing price in Boao for this study, but it may substantially decrease in the next few years as with anticipation of national approval by 2023. At present, anti-tumor drugs must go through national medical insurance negotiations to enter the Chinese medical insurance formulary. In 2022, the average price reduction of 67 drugs upon entering the national medical insurance formulary was 61.71%. In the previous 3 years, the price reductions were 56.7, 60.7, and 53.8% respectively through negotiations led by the National Healthcare Security Administration. Considering the price of T-Dxd may drastically decreased when it enters the Chinese medical insurance formulary, the minimum cost of T-Dxd was set to a 50% decrement from the baseline value in the one-way sensitivity analysis. However, even with the 50% price decrease, the resulting ICER of $162,768.63 per QALY was still much higher than the preset WTP.

WTP is a critical parameter to determine whether the treatment is cost-effective. When the ICER was lower than the WTP, the treatment was considered to be favorably cost-effective. Currently, the WHO standard of WTP setting at 1–3 times GDP per capita is still widely used (30, 31). However, some studies have suggested that three times of GDP per capita is too high for WTP (32, 33). For patients at end of life, the National Institute of Health and Clinical Excellence (NICE) have raised the WTP threshold for life-extending treatments that are not considered cost-effective with conventional WTP (34). At present, there is lack of effective treatment for HER2-low metastatic breast cancer refractory to standard treatment. The expected survival of these patients is < 24 months, and T-Dxd could extend their survival time by more than 3 months comparing to single-agent chemotherapy. Therefore, we chose a high WTP threshold based on the NICE standard. But even if a high WTP is set, the results of this study showed that T-Dxd is still not cost-effective. Additionally, due to a series of new policies such as national centralized drug procurement and national medical insurance negotiations, the prices of anti-cancer drugs in China have greatly reduced in recent years. Therefore, in addition to the predicted price reduction of T-Dxd, the cost of alternative chemotherapy is also expected to decline, which may trigger the ICER to increase even higher. The results of PSA demonstrated that T-Dxd had no chance in practice to be cost-effective at the current payment threshold in China.

There are some limitations with this model-based cost-effectiveness analysis. Imprecise estimates and assumptions were made where it was necessary. First, the one-way sensitivity analysis showed the assumed cost of T-Dxd had significant impact on the results, but the T-Dxd price may drastically fluctuate in the next few years upon national approval. Secondly, DESTINY-Breast04 did not provide the information about the utility scores of the PFS and PD, thus the utility value referenced in this study was not based on Chinese population. Moreover, DESTINY-Breast04 only reported the AE rates for all patients. We hypothesized that the AE incidences were similar among the hormone receptor–positive cohort and all patients, thereby the cost of AEs was estimated based on the AE incidences of all patients. As of December 2022, T-Dxd has not yet been approved for marketing in Chinese mainland; therefore, we performed model-based cost-effectiveness analyses based on the RCT DESTINY-Breast04, the results of which may deviate from real world experience. As a result, imprecise estimates and assumptions were inevitable. The robustness was measured using sensitivity analysis and the results of sensitivity analyses showed that the results were stable.

In conclusion, although T-Dxd in previously treated HER2-low advanced breast cancer showed excellent clinical efficacy, the results of our study suggested that T-Dxd, comparing with single agent chemotherapy, was not cost effective from the perspective of the Chinese healthcare system. Another drug of the ADC class called T-DM1 proactively reduced its price by 50% after T-Dxd filed its application for marketing, therefore T-Dxd may need to mark down its price by a huge degree to appear cost-effective.

## Data availability statement

The original contributions presented in the study are included in the article/supplementary material, further inquiries can be directed to the corresponding author.

## Ethics statement

Ethical review and approval was not required for the study on human participants in accordance with the local legislation and institutional requirements. Written informed consent for participation was not required for this study in accordance with the national legislation and the institutional requirements.

## Author contributions

Data curation: MZ, ZH, and HZ. Formal analysis: MZ, XX, and FW. Methodology: TX and FW. Writing—original draft: MZ and ZH. Writing—review and editing: FW. All authors contributed to the article and approved the submitted version.
